# Beyond platinum: synthesis, characterization, and *in vitro *toxicity of Cu(II)-releasing polymer nanoparticles for potential use as a drug delivery vector

**DOI:** 10.1186/1556-276X-6-445

**Published:** 2011-07-11

**Authors:** Alesha N Harris, Barbara R Hinojosa, Montaleé D Chavious, Robby A Petros

**Affiliations:** 1 Department of Chemistry, University of North Texas, 1155 Union Circle, CB#305070, Denton, TX, 76203-5017, USA

**Keywords:** copper, polymer nanoparticles, copper ion release, drug delivery, oxidative stress, HeLa cells

## Abstract

The field of drug delivery focuses primarily on delivering small organic molecules or DNA/RNA as therapeutics and has largely ignored the potential for delivering catalytically active transition metal ions and complexes. The delivery of a variety of transition metals has potential for inducing apoptosis in targeted cells. The chief aims of this work were the development of a suitable delivery vector for a prototypical transition metal, Cu^2+^, and demonstration of the ability to impact cancer cell viability via exposure to such a Cu-loaded vector. Carboxylate-functionalized nanoparticles were synthesized by free radical polymerization and were subsequently loaded with Cu^2+ ^via binding to particle-bound carboxylate functional groups. Cu loading and release were characterized via ICP MS, EDX, XPS, and elemental analysis. Results demonstrated that Cu could be loaded in high weight percent (up to 16 wt.%) and that Cu was released from the particles in a pH-dependent manner. Metal release was a function of both pH and the presence of competing ligands. The toxicity of the particles was measured in HeLa cells where reductions in cell viability greater than 95% were observed at high Cu loading. The combined pH sensitivity and significant toxicity make this copper delivery vector an excellent candidate for the targeted killing of disease cells when combined with an effective cellular targeting strategy.

## Introduction

The field of drug delivery focuses primarily on delivering small organic molecules or DNA/RNA as therapeutics and has largely ignored the potential for delivering catalytically active transition metal ions and complexes [1-3]. Some success has been realized in the case of cisplatin [4-7]; however, vectors designed to deliver other metal species are rare [8-11]. Thus, a significant opportunity exists for examining the impact of selectively delivering a variety of metal ions and complexes to cells. Rational design of a vector capable of sequestering and releasing metals is therefore needed. Nanoparticles based on nanoscale metal/organic frameworks and infinite coordination polymers are being pursued actively as drug delivery vectors; however, the metal is used as a structural component of the particle, and in general is not the therapeutically active moiety [12,13]. We have developed a prototypical approach that allows us to accomplish reversible metal binding to polymeric nanoparticles that are stable in aqueous solutions and that are capable of releasing bound metal in a pH-dependent manner. We also postulate that release could be triggered by a change in reduction potential. Sensitivity to pH allows one to capitalize on the drop in pH known to occur along the endosomal/lysosomal pathway for endocytosis to facilitate release, while sensitivity to a reducing environment could stimulate release in response to the reducing nature of cytosol [1].

If targeted delivery can be achieved, transition metal species would be expected to display a range of activities inside the cell ranging from redox catalysis to the targeted binding of biomolecules [14-17]. Recent findings [18-26] indicate that many types of nanoparticles are capable of inducing oxidative stress, which is of great concern in terms of the nanotoxicology of particles being pursued for a variety of consumer products. Furthermore, some colloidal metal particles have been shown to be particularly effective at generating reactive oxygen species (ROS) presumably through the slow leaching of metal ions from the particle core [19-21,25]. Increased ROS production is capable of inducing biological damage and has been linked to a variety of disease states including cancer, cardiovascular disease, arthritis, diabetes, Alzheimer's disease, and Parkinson's disease [27]. Cancer cells use ROS to suppress apoptosis, accelerate proliferation, induce metastasis and angiogenesis, and promote genetic instability through DNA damage [27-32]. However, the inherent toxicity of increased ROS production represents an opportunity if it can be harnessed by selectively targeting ROS-generating particles to diseased cells [28,30]. In this case, it would be desirable to release large amounts of metal ions in a short period of time, which is opposite to what is observed for the slow leaching of metal ions from colloidal metal particles. Increased ROS production has the potential to induce cell death by altering the expression of apoptosis-related genes, such as Fas, *c-fos*, *c-jun*, *p*53, and Bcl-2 [22,24,33,34]. It is important to note that most chemotherapeutics display high levels of toxicity, and that their maximum tolerated dose is often dictated by the maximum tolerable off-target toxicity. Transition metal complexes also routinely exhibit high levels of toxicity; however, such toxicity does not limit their potential for treating disease [17]. For example, a series of Cu^2+ ^-containing compounds that exhibit high levels of cytotoxicity and genotoxicity are being actively pursued as cancer chemotherapeutics [35,36].

We have therefore designed a carboxylate-functionalized, polymer-based nanoparticle capable of sequestering a prototypical metal, Cu^2+^, for the ultimate goal of delivering Cu^2+ ^to cancer cells to facilitate apoptosis. The particles described here represent a single example of a multitude containing other metal/ligand combinations that can be envisaged [37]. Here, we report the synthesis, characterization, and metal binding properties of our Cu-binding particles, as well as preliminary *in vitro *toxicity in cancer cells

## Results

### Synthesis and characterization of Cu-loaded polymeric nanoparticles

Carboxylate-functionalized, acrylate-based nanoparticles were synthesized via standard microwave-assisted, free radical polymerization techniques [38]. Nanoparticles used for all experiments described in this work were prepared from an aqueous pre-polymer solution containing 50 wt.% of an acrylic acid monomer. Nanoparticles were synthesized in aqueous solution and remained well dispersed over the course of several weeks. Excess unreacted monomer was removed via dialysis and nanoparticle concentration was determined by lyophilizing a sample of purified particles and weighing the resultant solid.

**Figure 1:**
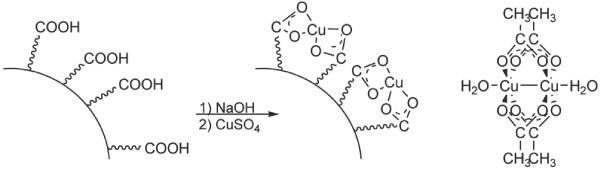
**Cu-loading chemistry for CuCNPs (left) and the structure of dinuclear Cu_2_(OAc)_4_(H_2_O)_2 _(right)**.

**Figure 2:**
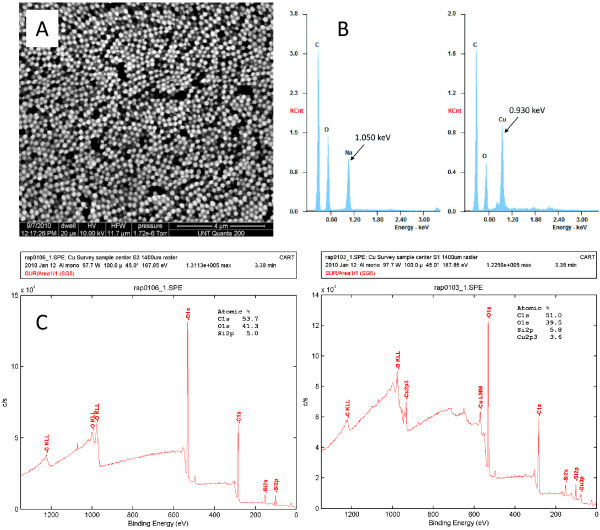
**Characterization of CuCNPs**. (**A**) SEM image of CuCNPs, (**B**) EDX spectra of CNPs before (left) and after (right) addition of Cu, (**C**) XPS spectra for CuCNPs (right) and control particles containing no Cu (left).

Cu^2+ ^loading to form Cu-loaded carboxylate-functionalized nanoparticles (CuCNPs) was accomplished by first adjusting the pH of the particle-containing solution to 7.0 using 1.0 M NaOH, which deprotonated the carboxylic acid groups, followed by the addition of CuSO_4 _in a 1:1 molar ratio to NaOH (Figure 1). The representation shown in Figure 1 for Cu binding to CuCNPs represents a mononuclear complex; however, a dinuclear complex like that observed for molecular copper acetate (also shown in Figure 1) is equally likely (note: the schematic in Figure 1 shows carboxylic acid groups only on the surface of the particle; however, the particle is a porous hydrogel, which allows copper to freely diffuse throughout the polymer network and bind to carboxylate groups on the interior of the particle as well). The particle solution was then dialyzed to remove unbound copper. Particle size of approximately 215 nm in solution was determined via dynamic light scattering (see Additional file 1) and a scanning electron microscope (SEM) image of dried CuCNPs is shown in Figure 2A.

The amount of Cu bound to CuCNPs was investigated using several analytical techniques including: inductively coupled plasma mass spectrometry (ICP MS), X-ray photoelectron spectroscopy (XPS), and energy-dispersive X-ray analysis (EDX). For ICP MS studies, the amount of unbound copper released during purification by dialysis was monitored for 48 h from a sample containing a known mass of particles (see Additional file 2). The difference between the amount released at 48 h and that contained in the original loading solution determined Cu loading, resulting in values ranging between 12 and 16 wt.% based on these reactions conditions. XPS was used to further confirm Cu loading and to probe Cu coordination sphere (Figure 2C). Cu weight percent measured by XPS was 15 wt.%; one of the peaks in the spectrum (933.9 eV) was consistent with that of a copper acetate complex.

Only peaks for C, O, and Cu were observed in EDX spectra obtained for CuCNPs (Figure 2B), and the measured weight percents were consistent with both ICP MS and XPS. EDX was also performed on samples immediately before and after the addition of CuSO_4_. Before CuSO_4 _treatment, only peaks for C, O, and Na were observed; after treatment, the Na peak disappeared and a Cu peak appeared. The amount of Cu^2+ ^loaded in CuCNPs could be varied trivially by adding a sub-stoichiometric amount of CuSO_4_. CuCNPs with 16, 12, 5, and 3 wt.% Cu were synthesized in this manner, and loading quantified via ICP MS.

### Cu release from CuCNPs

**Figure 3:**
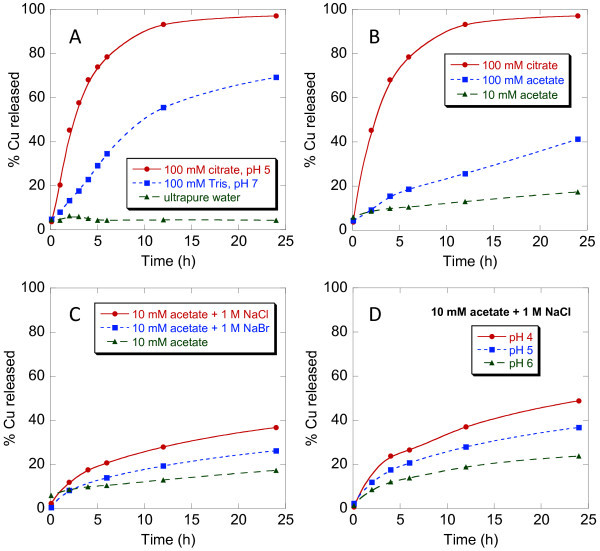
**Release of Cu under various reaction conditions**. (**A**) Initial release data simulating endosome/lysosome pH conditions, (**B**) release as a function of buffering species, (**C**) release as a function of added competing ligand, (**D**) release as a function of pH in the absence of competing ligand effects.

The applicability of CuCNPs for triggered release [1] has been studied by examining the rate of Cu release in response to changes in pH. Three identical samples of purified CuCNPs were dialyzed in either ultrapure water, 100 mM TRIS buffer at pH 7, or 100 mM citrate buffer at pH 5 and the release of Cu was monitored for 48 h by ICP MS (Figure 3A). Virtually no release was observed in ultrapure water with approximately 95% of the loaded Cu remaining bound to the particles over the course of the experiment. Cu release was observed at pH 7; however, release was much slower at this pH (particles at pH 7 had released approximately 55% of their Cu at 12 h) compared to pH 5. At pH 5, CuCNPs had released over 93% of their loaded Cu at 12 h, and at 48 h, complete release was observed. Cu weight percents determined by ICP MS at the end of this set of experiments were 12.1, 1.7, 0.0 wt.% Cu for CuCNPs dialyzed in ultrapure water, pH 7 buffer, and pH 5 buffer, respectively.

Qualitatively, a color change was observed in CuCNPs upon release of Cu where the particle color gradually turned from blue to white. CuCNPs dialyzed at pH 5 turned white within 12 h; whereas, those dialyzed at pH 7 remained faintly blue even at the end of 48 h. Particles were then collected from the dialysis tubing and analyzed further for Cu content by EDX. Cu weight percents were 12.7, 3.3, and 0.7 for particles in ultrapure water, pH 7, and pH 5, respectively, consistent with ICP MS data. Elemental analysis by an outside vendor of CuCNPs dialyzed in ultrapure water (Cu wt.% = 10.92) and at pH 5 (Cu wt.% = 0.04) further confirmed our experimental findings. Table 1 contains a summary of Cu weight percents determined for each sample by the various the experimental methods employed.

**Table 1:** Cu content (in weight percent) for CuCNPs used in Cu release experiments

	ICP MS	EDX	Elemental analysis
**Ultrapure water**	12.1	12.7	10.92
**pH 7 buffer**	1.7	3.3	Not measured
**pH 5 buffer**	0.0	0.7	0.04

The lack of release in ultrapure water compared to buffered solutions implied that competing ligands other than water must be present in order to facilitate Cu release, which led us to further investigate Cu release as a function of competing ligand. Cu release experiments conducted in 100 mM citrate buffer at pH 4, 5, and 6 revealed faster metal release at pH 6 than at pH 4 (see Additional file 3), which was surprising and further illustrated that metal release is affected by more than just pH. Here, the differences in rates can be attributed to differences in concentrations of the various species present in the buffer as the pH is lowered (see Discussion). Figure 3B shows that Cu release was accelerated in 100 mM citrate buffer compared to 100 mM acetate buffer at pH 5 also implying that the conjugate base of the buffering species plays an important role. Buffer strength also influenced Cu release rates as can also be seen in Figure 3B (10 and 100 mM acetate buffer at pH 5). Cu release rate in 10 mM acetate buffer at pH 5 increased upon the introduction of an appropriate competing ligand, such as chloride (Figure 3C). The identity of the competing ligand that was added also influenced the rate of Cu release as was observed on substituting bromide for chloride (Figure 3C). In the absence of competing ligand effects (see Discussion), Cu release displayed pH-dependent behavior with faster release being observed as the pH was lowered from 6 to 4 (Figure 3D). These combined experiments illustrate both pH- and competing ligand-dependent effects on the rate of Cu release (see Discussion).

### *In vitro *toxicity of CuCNPs in HeLa cells

**Figure 4:**
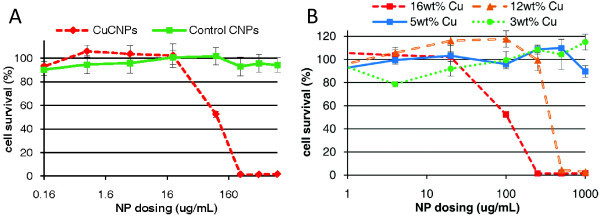
***In vitro *toxicity of CuCNPs**. (**A**) HeLa cell viability as measured via an assay based on MTT at 48 h showing toxicity only after the addition of Cu to the nanoparticles, (**B**) a similar experiment showing the reduced toxicity of CuCNPs upon reduction of Cu content.

The *in vitro *toxicity of CuCNPs in HeLa cells (a cervical adenocarcinoma) was investigated via an assay based on the MTT reagent (3-(4,5-dimethylthiazol-2-yl)-2,5-diphenyl-tetra-zolium bromide). Particles were added to wells with cells at the desired particle concentrations; the plates were incubated for 48 h, followed by an assessment of cell survival via the MTT reagent. Control particles (particles without added Cu) showed no toxicity up to the highest dosing. In contrast, Cu-loaded particles displayed significant toxicity with an IC_50 _of approximately 100 μg/mL (Figure 4A). The toxicity of free copper acetate was measured to allow for direct comparison with the amount of Cu contained in CuCNPs (see Additional file 4). We found that Cu contained in CuCNPs was significantly more toxic than an equivalent amount of free Cu dose, implying that the observed Cu toxicity was particle mediated. Finally, the amount of Cu loaded in CuCNPs was varied and its effect on toxicity investigated (Figure 4B). CuCNPs became significantly less toxic as the Cu loading was reduced, with little or no toxicity being observed for CuCNPs containing 3, or 5 wt.% Cu.

## Discussion

### Synthesis and characterization of Cu-loaded polymeric nanoparticles

A prototypical approach for sequestering and releasing metal ions from a delivery vector has been demonstrated. In the current example, Cu^2+ ^was loaded to acrylate-based nanoparticles with Cu loadings as high as 16 wt.%. This strategy relied on functionalizing the nanoparticle with carboxylate ligands to bind Cu^2+^; however, other metal/ligand/polymer combinations could be synthesized including those employing other polymers routinely used in targeted drug delivery, such as PLGA, chitosan, or dextran. Thus, the metal/ligand chemistry is readily adaptable to, and independent from, the desired polymeric material used as the delivery vector. The rational design of carriers to deliver other metal species should be possible using this approach.

### Cu release from CuCNPs

**Figure 5:**
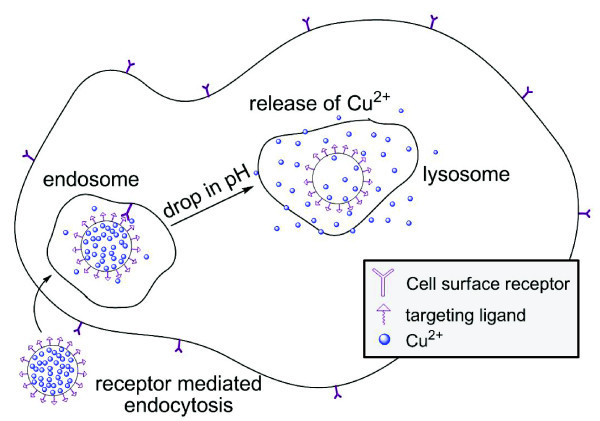
**Proposed intracellular release mechanism based on pH**.

The loading and stimuli-responsive release of transition metals and any drug molecule in general from a delivery are major factors that ultimately determine the success or failure of that vector when applied to targeted drug delivery. One of the goals of this work was to demonstrate that CuCNPs were capable of responding to changes in pH to facilitate Cu release. A general schematic for the expected *in vitro *behavior is shown in Figure 5 (targeting ligands were not used in the experiments described here, but will be incorporated in the future). Initial Cu release experiments were conducted at pH 5 and 7 to mimic conditions that would be present during endocytosis of the nanoparticle along an endosomal pathway. Those experiments (Figure 3A) were promising and showed release to be much faster at pH 5 vs. pH 7, which would trigger Cu release upon particle internalization. It was postulated that protonation of the carboxylate groups on the nanoparticle would reduce the binding affinity of the ligand for Cu thereby facilitating release. Somewhat surprisingly, however, CuCNPs released virtually no Cu in ultrapure water, which has a neutral to slightly acidic pH. While this feature is promising in terms of the stability of solutions of CuCNPs over long periods of time, Cu release cannot simply be a function of pH but must also depend on the presence of ligands that can compete with the particle-bound carboxylate groups in Cu binding. This feature led to a series of additional experiments to elucidate the effect of competing ligands on Cu release with the idea that the underlying principles governing release could be used in the design/optimization of this class of delivery vectors.

Release of Cu from CuCNPs in citrate buffer at pH 4, 5, and 6 (see Additional file 3) illustrated the effect of competing ligand concentration on the rate of Cu release, which was actually faster at pH 6 compared to pH 4. This can be explained by looking at the various protonation states of citrate as a function of pH to determine the competing ligands present in solution. Equations 1-4 were used to determine the relative concentrations of L^3-^, LH^2-^, LH_2_^-^, and LH_3 _(L = citrate) using pK_a _values of 3.13, 4.76, and 6.40 for citric acid (*K*_1 _= 7.40 × 10^-4^, *K*_2 _= 1.70 × 10^-5^, *K*_3 _= 4.00 × 10^-7^). The relative concentrations of L^3-^, LH^2-^, LH_2_^-^, and LH_3 _are 27%, 69%, 4%, and 0% at pH 6 while at pH 4 the relative concentrations for the same species are 0%, 13%, 77%, and 10%. At low pH, the predominate species is LH_2_^- ^whereas at high pH the predominate species is LH^2- ^with a substantial amount of L^3- ^being present as well. One would expect the affinity of these ligands for Cu to increase with increasing negative charge, and that is clearly what is observed. So, even though the particle-bound carboxylate is protonated to a lesser extent at pH 6, the presence of the di- and tri-anion form of citrate effectively compete out Cu.(1)(2)(3)(4)

In an effort to decouple competing ligand effects due to the presence of changing buffering species with actual pH-dependent Cu release, we sought to reduce the buffer effect while concomitantly introducing competing ligands that were unaffected by solution pH. The use of citrate buffer was less than ideal in this case due to the multiple acidic protons capable of generating four possible species in solution. The use of acetate buffer in place of citrate was expected to reduce this complexity. Acetate buffer generates only two species with relative concentrations of A^- ^and HA being 95% and 5% at pH 6 and 15 and 85% at pH 4, respectively (pK_a _= 4.75). Furthermore, even though the concentration of the anion changes in this case as well, and is higher at pH 6, this species is identical to the particle-bound carboxylate groups making it a less effective competitor compared to the species present in citrate buffer at the same pH. A direct comparison of Cu release at pH 5 in acetate buffer and citrate buffer (Figure 3B), both at 100 mM, confirmed this assumption. Cu release was much slower in acetate buffer most likely a result of the reduced competitive nature of A^- ^compared to LH^2-^. Cu release in acetate buffer could be further reduced by lowering the buffer strength to 10 mM (Figure 3B).

With buffer effects reduced, a competing ligand was then introduced. Chloride was chosen as an appropriate competing ligand because its concentration would not be effected by the pH of the solutions being investigated. Indeed, the introduction of chloride increased the rate of Cu release in acetate buffer (Figure 3C). Again, Cu release was highly dependent on the identity of competing ligand as illustrated by comparing the effect of added chloride vs. bromide (Figure 3C). Next, Cu release was monitored in 10 mM acetate buffer at pH 4, 5, and 6 containing a large excess of chloride (1 M NaCl, Figure 3D). With changes in competing ligand concentrations effectively minimized, pH-dependent Cu release was clearly demonstrated and release was accelerated as the pH was reduced.

### *In vitro *toxicity of CuCNPs in HeLa cells

The toxicity of CuCNPs to cancer cells was investigated and results demonstrated that the delivery vector itself, particles containing no Cu, was not toxic and that CuCNPs displayed significant toxicity depending on dosing (Figure 4A). Furthermore, the delivery of Cu contained in CuCNPs was more toxic than an equivalent amount of free Cu dose implying that the phenomenon was particle mediated (see Additional file 4). This effect is likely attributable to the differences in modes of internalization for the two forms of Cu. Free copper would be taken up by the cell via normal metal trafficking pathways that utilize metal-binding proteins located on the cell surface. As the cell begins to experience metal overload, those receptors would be internalized and degraded to prevent further metal accumulation. CuCNPs would be expected to be internalized by an entirely different pathway that is not subject to the normal cellular mechanisms for controlling metal homeostasis. Thus, the cell's normal metal overload defenses were likely bypassed leading to unregulated Cu uptake. As the Cu-loading in CuCNPs was reduced, cell survival improved with little or no toxicity being observed for CuCNPs containing 3 or 5 wt.% Cu (Figure 4B). The most likely source of toxicity was induced oxidative stress (see Introduction) and future experiments will probe the mechanism of cell death to determine the validity of this hypothesis.

## Conclusions

In summary, we have synthesized Cu-loaded polymeric nanoparticles that release bound Cu in a pH-dependent manner. Cu loading and release were characterized by several analytical techniques where we demonstrated the ability to load up to 16 wt.% Cu. The release of bound Cu from CuCNPs was found to be both pH and competing ligand dependent. Decoupling these effects was non-trivial, but was accomplished through careful selection of reaction conditions. Based on the behavior observed, we conclude that simple protonation of the particle-bound carboxylate, while rate accelerating was not sufficient to promote release rather the presence of a ligand capable of displacing the carboxylate was required. The complexities described here will undoubtedly increase dramatically when CuCNPs are introduced to biologically relevant media containing a plethora of potential ligands. Our coupling strategy allows us to capitalize on the pH gradient observed along the endosome/lysosome pathway for particle internalization for targeted delivery of Cu [1]. CuCNPs were capable of inducing toxicity in cancer cells where reductions of >95% viability were observed at high Cu loadings. The stimuli-responsive release and toxicity of Cu in CuCNPs meets the requirements for application in targeted drug delivery.

## Methods

### General considerations

Methyl methacrylate, acrylic acid, and poly(ethylene glycol) (n) diacrylate (*n *= 200 = MW of PEG block) were purchased from Polysciences, Inc. (Warrington, PA, USA) and used as received. Potassium persulfate, copper sulfate, copper acetate, nitric acid (trace metal grade) were from Fisher Scientific (Pennsylvania, PA, USA). All materials were used as received unless otherwise noted. Microwave reactions were conducted in a Synthos 3000 from Anton Paar (Ashland, VA, USA). ICP MS experiments were conducted on a Varian 820-MS (Varian Inc., Lake Forest, CA, USA) using the following parameters: plasma flow 17.5 L/min, auxiliary flow 1.65 L/min, sheath gas 0.13 L/min, and nebulizer flow 0.89 L/min. The torch alignment had a sampling depth of 5 mm. The RF power was set at 1.3 kW. The pump rate was 3 rpm, and the stabilization delay was 30 s. The ion optics parameters were: first extraction lens -1 V, second extraction lens -191 V, third extraction lens -206 V, corner lens -236 V, mirror lens left 56 V, mirror lens right 49 V, mirror lens bottom 16 V, entrance lens 0 V, fringe bias -2.5 V, entrance plate -31 V, and pole bias 0 V. CRI parameters were skimmer gas off, sampler gas off, skimmer flow 0 mL/min, and sampler flow 0 mL/min. ICP MS tubing was rinsed in between samples to avoid sample contamination.

### Synthesis of nanoparticles

An aqueous solution (58.8 mL) containing acrylic acid (0.57 g), methyl methacrylate (0.575 g), PEG diacrylate (0.053 g), and potassium persulfate (0.164 g) was prepared in a PTFE vessel for a Synthos 3000 16MF100 rotor in a freshly regenerated inert atmosphere glovebox. The vessel was sealed, removed from the glovebox, and placed in the 16MF100 rotor along with seven other vessels containing 60 mL of water each. The rotor was placed in the microwave and then heated to 90°C for 60 min with a maximum microwave power of 1400 W (see Additional file 5). The internal temperature and pressure of the vessel containing the monomer solution were monitored via a p/T sensor accessory (Anton Paar). The resulting nanoparticle solution was dialyzed in 4 L of ultrapure water for 48 h with a change in the water after the first 24 h. The particle concentration after purification was determined by lyophilizing a known volume and then weighing the resulting solid, which resulted in a final particle concentration of 12.8 mg/mL. Based on this number, a total of 0.896 g of particles was synthesized with approximately 75% conversion of monomer to particles.

### Copper loading

A 3-mL aliquot of the nanoparticle solution was adjusted to a pH of 7 using NaOH followed by the addition of copper sulfate in a 1:1 molar ratio with amount of NaOH added. The particle solution was then dialyzed in 1.5 L of ultrapure water for 48 h to remove unbound copper. Particle size of approximately 215 nm was determined via dynamic light scattering (DLS).

### ICP MS Cu-loading studies

For Cu-loading studies, the Cu-loading solution containing CuSO_4 _and nanoparticles was dialyzed in 1.5 L of ultrapure water. Samples (1 mL each) were removed at 1, 2, 3, 4, 5, 6, 12, 24, and 48 h, diluted in 1% nitric acid and then analyzed for^63^Cu content via ICP MS. Cu content was determined by comparison with a calibration curve generated using known samples (see Additional file 6).

### ICP MS Cu release studies

Purified CuCNP-containing solutions (3 mL) were dialyzed in 1.5 L of the desired buffering solution. Samples (1 mL each) were removed at 0.08, 1, 2, 3, 4, 5, 6, 12, 24, and 48 h, diluted in 1% nitric acid and then analyzed for^63^Cu content via ICP MS. Cu content was determined by comparison with a calibration curve generated using known samples.

### X-ray photoelectron spectroscopy

XPS spectra were acquired with a PHI 5000 VersaProbe™ Scanning XPS Microprobe (Physical Electronics Inc., Chanhassen, MN, USA). Samples were prepared by spotting 5 μL of the desired particle-containing solution onto a glass slide and then drying under vacuum.

### Scanning electron microscopy and energy-dispersive X-ray analysis

SEM images and EDX spectra were obtained with a Quanta ESEM microscope (FEI, Hillsboro, OR, USA) equipped with a Sapphire Si(Li) detecting unit for EDX (EDAX Inc., Mahwah, NJ, USA). Samples were prepared by spotting 5 μL of the desired particle-containing solution onto a glass slide, drying under vacuum, and then repeating the spot/dry three times to produce samples with enough thickness to prevent interference from the glass slide during EDX analysis. Samples were then coated with Au (2-5 nm thickness) using a Cressington 108 Manual Sputter Coater (Ted Pella, Redding, CA, USA). Images were obtained with an acceleration voltage of 5-15 kV and EDX spectra were obtained with an acceleration voltage of 5 kV.

### Elemental analysis

Microanalysis was performed by Columbia Analytics (formerly Desert Analytics) in Tucson, AZ. Samples (100 mg) for elemental analysis were prepared by lyophilizing the desired nanoparticle-containing solution, which were further dried for 4 h at 25°C under vacuum prior to analysis.

### Cell viability measurements

HeLa cells were purchased from ATCC (cat. # CCL-2), and maintained in Eagle's Minimum Essential Medium (ATCC, cat. # 30-2003) with 10% FBS (Thermo Scientific HyClone, South Logan, UT, USA). Five thousand cells per well seeded on 96-well plates and incubated overnight at 37°C (5% CO_2_). The desired particle amounts were added to the wells and the plates were incubated for an additional 48 h at 37°C (5% CO_2_). After the incubation, cell viability was evaluated with the MTT reagent. Media was removed each well and replaced with fresh media containing 1 mg/mL MTT. The cells were incubated for 4 h at 37°C (5% CO_2_) after which time the media was removed and replaced with DMSO. Light absorption was measured on a Synergy 2 multi-mode microplate reader (BioTek, Winooski, VT, USA). The viability of the cells exposed to particles was expressed as a percentage of the viability of cells grown in the absence of particles on the same plate.

## Competing interests

The authors declare that they have no competing interests.

## Authors' contributions

AH carried out Cu loading and release studies via ICP MS, participated in the design and coordination of ICP MS studies, and helped draft the manuscript. BH optimized microwave conditions for the free radical polymerization reaction used in the synthesis of polymeric nanoparticles. MC carried out particle synthesis, and metal loading. RP conceived of the study, and participated in its design and coordination, carried out *in vitro *toxicity studies, SEM and EDX analysis and drafted the manuscript.

## Supplementary Material

Additional file 1 **DLS results for purified CuCNPs**. graph showing particle size as determined by Dynamic Light Scattering. Click here for file

Additional file 2 **Release of unbound Cu over time during purification of CuCNPs as monitored by ICP MS**. graph showing all copper that is not bound to the particle is removed by dialysis for 48 h. Click here for file

Additional file 3 **Release of Cu from purified CuCNPs over time in 100 mM citrate buffer at pH 4, 5, and 6**. graph showing that Cu release is actually slower as the pH is lowered due to competing ligand effects. Click here for file

Additional file 4 ***In vitro *toxicity for comparison of Cu in CuCNPs *versus *similar dosing of free Cu(OAc)_2_**. graph showing copper contained in nanoparticles was more toxic than an equivalent amount of copper dosed as a free complex. Click here for file

Additional file 5 **Graph of reaction time vs. temperature, pressure, and microwave power during nanoparticle synthesis**. graphs showing microwave conditions used for nanoparticle synthesis. Click here for file

Additional file 6 **Typical calibration curve used for determining the Cu concentration in unknown samples**. calibration curve generated from samples containing a known amount of copper. Click here for file
