# Supplementary figures and images for: Beyond platinum: synthesis, characterization, and in vitro toxicity of Cu(II)-releasing polymer nanoparticles for potential use as a drug delivery vector
Source: Nanoscale Res Lett. 2011 Jul 11;6(1):445. doi: 10.1186/1556-276X-6-445 (PMC3211864; doi:10.1186/1556-276X-6-445)

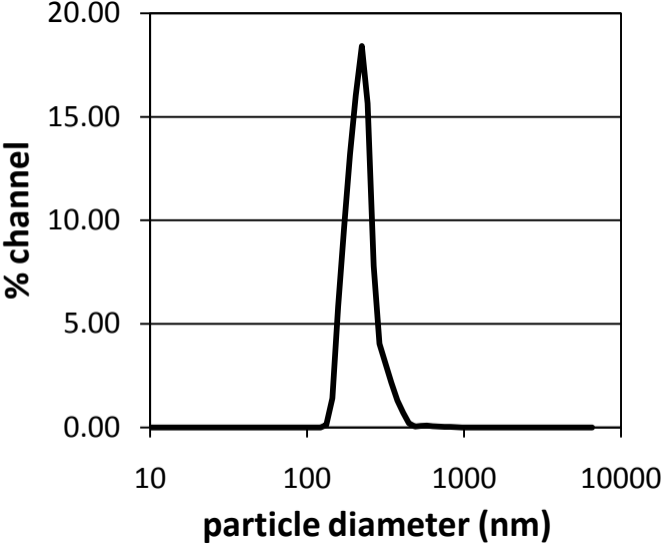

Supplement: Additional file 1 — DLS results for purified CuCNPs. graph showing particle size as determined by Dynamic Light Scattering. [file 1556-276X-6-445-S1.PDF]

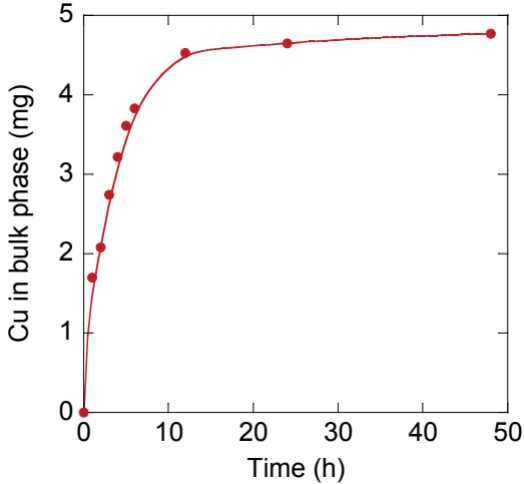

Supplement: Additional file 2 — Release of unbound Cu over time during purification of CuCNPs as monitored by ICP MS. graph showing all copper that is not bound to the particle is removed by dialysis for 48 h. [file 1556-276X-6-445-S2.PDF]

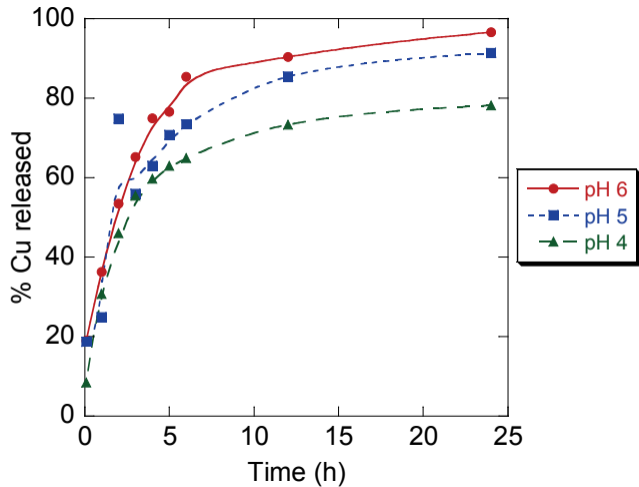

Supplement: Additional file 3 — Release of Cu from purified CuCNPs over time in 100 mM citrate buffer at pH 4, 5, and 6. graph showing that Cu release is actually slower as the pH is lowered due to competing ligand effects. [file 1556-276X-6-445-S3.PDF]

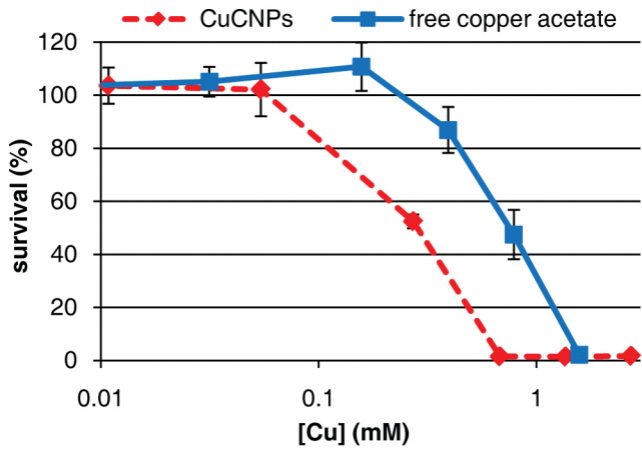

Supplement: Additional file 4 — In vitro toxicity for comparison of Cu in CuCNPs versus similar dosing of free Cu(OAc)2. graph showing copper contained in nanoparticles was more toxic than an equivalent amount of copper dosed as a free complex. [file 1556-276X-6-445-S4.PDF]

— Pressure — Temperature — Power

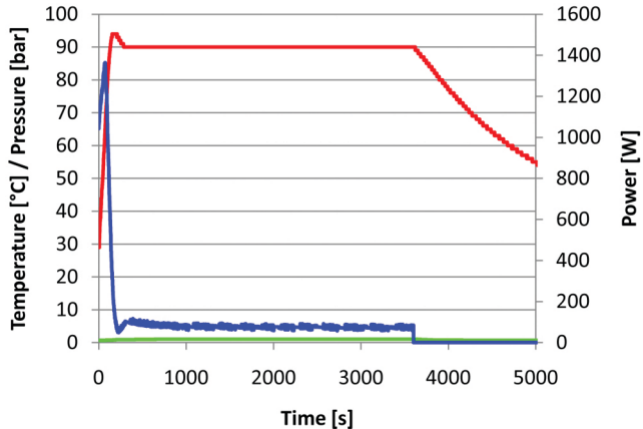

Supplement: Additional file 5 — Graph of reaction time vs. temperature, pressure, and microwave power during nanoparticle synthesis. graphs showing microwave conditions used for nanoparticle synthesis. [file 1556-276X-6-445-S5.PDF]

# Avg Signal for Cu63

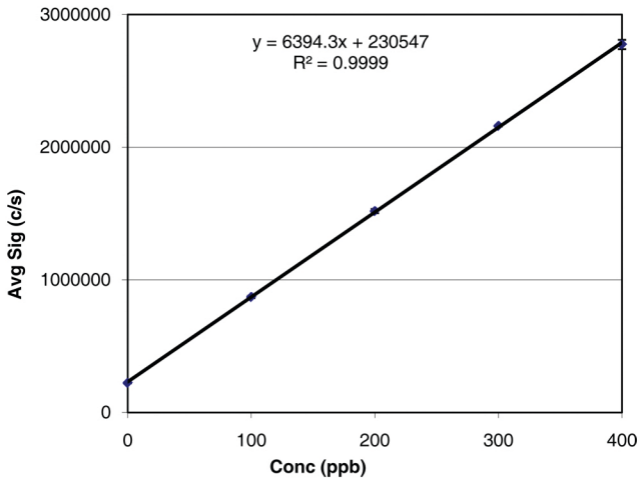

Supplement: Additional file 6 — Typical calibration curve used for determining the Cu concentration in unknown samples. calibration curve generated from samples containing a known amount of copper. [file 1556-276X-6-445-S6.PDF]
